# Interruption of mycothiol synthesis and intracellular redox status impact iron-regulated reporter activation in *Mycobacterium smegmatis*

**DOI:** 10.1128/spectrum.00487-24

**Published:** 2024-06-11

**Authors:** Alexandra H. Miller, Frances Marks, Luming Chan, Helene Botella, Dirk Schnappinger, Sabine Ehrt

**Affiliations:** 1 Department of Microbiology and Immunology, Weill Cornell Medical College, New York, New York, USA; 2 Immunology and Microbial Pathogenesis Graduate Program, Weill Cornell Graduate School of Medical Sciences, Cornell University, New York, New York, USA; 3 Biochemistry and Structural Biology, Cell and Developmental Biology, and Molecular Biology Program, Weill Cornell Graduate School of Medical Sciences, Cornell University, New York, New York, USA; University at Albany, Albany, New York, USA

**Keywords:** *Mycobacterium*, promoters, iron regulation, mycothiol

## Abstract

Iron scavenging is required for full virulence of mycobacterial pathogens. During infection, the host immune response restricts mycobacterial access to iron, which is essential for bacterial respiration and DNA synthesis. The *Mycobacterium tuberculosis* iron-dependent regulator (IdeR) responds to changes in iron accessibility by repressing iron-uptake genes when iron is available. In contrast, iron-uptake gene transcription is induced when iron is depleted. The *ideR* gene is essential in *M. tuberculosis* and is required for bacterial growth. To further study how iron regulates transcription, wee developed an iron responsive reporter system that relies on an IdeR-regulated promoter to drive Cre and *loxP* mediated recombination in *Mycobacterium smegmatis*. Recombination leads to the expression of an antibiotic resistance gene so that mutations that activate the IdeR-regulated promoter can be selected. A transposon library in the background of this reporter system was exposed to media containing iron and hemin, and this resulted in the selection of mutants in the antioxidant mycothiol synthesis pathway. We validated that inactivation of the mycothiol synthesis gene *mshA* results in increased recombination and increased IdeR-regulated promoter activity in the reporter system. Further, we show that vitamin C, which has been shown to oxidize iron through the Fenton reaction, can decrease promoter activity in the *mshA* mutant. We conclude that the intracellular redox state balanced by mycothiol can alter IdeR activity in the presence of iron.

**IMPORTANCE:**

*Mycobacterium smegmatis* is a tractable organism to study mycobacterial gene regulation. We used *M. smegmatis* to construct a novel recombination-based reporter system that allows for the selection of mutations that deregulate a promoter of interest. Transposon mutagenesis and insertion sequencing (TnSeq) in the recombination reporter strain identified genes that impact iron regulated promoter activity in mycobacteria. We found that the mycothiol synthesis gene *mshA* is required for IdeR mediated transcriptional regulation by maintaining intracellular redox balance. By affecting the oxidative state of the intracellular environment, mycothiol can modulate iron-dependent transcriptional activity. Taken more broadly, this novel reporter system can be used in combination with transposon mutagenesis to identify genes that are required by *Mycobacterium tuberculosis* to overcome temporary or local changes in iron availability during infection.

## INTRODUCTION

Tuberculosis, caused by the communicable airborne pathogen *Mycobacterium tuberculosis*, is the leading cause of death from a bacterial infection worldwide. *M. tuberculosis* (Mtb) bacilli are inhaled and travel to the lower respiratory tract where they are phagocytosed by alveolar macrophages (1). During infection, the host suppresses mycobacterial replication and dissemination by enclosing bacteria within macrophage phagosomes. In this environment, mycobacteria have reduced access to numerous essential nutrients including iron, which is required to perform redox reactions important for DNA synthesis and cellular respiration (2). Mtb, and the related saprophyte *Mycobacterium smegmatis*, express proteins to acquire extracellular iron from the environment. Both species secrete iron chelators called siderophores to bind extracellular ferric iron and bring it into the bacterial cell. Mtb has two types of siderophore, the membrane associated mycobactin (MBT) and the soluble carboxymycobactin (cMBT) (3). Transcription of the genes required to synthesize MBT and cMBT is regulated by the iron-dependent regulator IdeR, which represses siderophore gene transcription when bound to iron and de-represses siderophore transcription in low iron conditions (4). *M. smegmatis* (Msm) can synthesize MBT and cMBT like Mtb, but also produces the siderophore exochelin, which is encoded by the *fxb* genes (5). Mutagenesis experiments have suggested that the exochelin system is essential in Msm MBT mutants and vice versa, and that the *esx-3* gene cluster which encodes a type VII secretion system is also important for iron acquisition (6, 7). In Msm that lacks both the MBT and exochelin uptake systems, the porin MspA and putative heme transporter MmpL11 become essential, suggesting that these two systems may also mediate iron transport (6). Loss of *mspA* activates IdeR-dependent genes under high-iron conditions, supporting a role for porins in iron uptake (8).

The IdeR protein regulates approximately one-third of the iron responsive genes in Mtb (9). IdeR is essential for growth of Mtb in culture, and unregulated iron uptake can increase oxidative stress, leading to DNA damage, protein damage and cell death (9, 10). Importantly, IdeR is also required for replication of Mtb in mice (10). There is evidence that oxidative stress can also regulate IdeR, as MBT synthesis genes are upregulated in response to nitric oxide stress (11). Iron is tightly controlled in mycobacteria and can induce oxidative stress through the generation of hydroxyl radicals via the Fenton reaction (12–14).

In contrast to the repressor function of IdeR, HupB acts as a positive regulator for siderophore synthesis in low iron (15). HupB binds the mycobactin B (*mbtB*) promoter in the presence of iron at concentrations lower than those required for IdeR binding, suggesting it may act as a positive regulator for *mbtB* transcription in reduced iron conditions. Mtb *hupB* mutants have reduced levels of both cMBT and MBT and transcriptomic data revealed that many iron-regulated genes are downregulated in the *hupB* mutant, including *mbtB* (15). HupB binds Fe^3+^ and subsequently interacts with the *mbtB* promoter region to induce transcription, but also acts as a ferritin-like iron storage protein in the presence of oxidized iron (16). Therefore, we hypothesize that the oxidative state of iron can affect the *mbtB* promoter through de-repression via IdeR and positive regulation via HupB.

To elucidate regulatory mechanisms of the iron-dependent MBT synthesis-encoding genes, we designed a recombination system that reports on promoter activity of the *mbtB* gene from Mtb, and includes both the IdeR and HupB regulatory regions. We expressed Cre recombinase from the *mbtB* promoter in Msm strains containing a zeocin resistance gene (*bleoR*). In these strains, the transcription of *bleoR* depends on the excision of a *loxP*-flanked terminator that is inserted between *bleoR* and its promoter. *MbtB* promoter activity leads to expression of Cre, which mediates terminator excision and results in zeocin resistant Msm that can be selected for on agar plates. This method can be used to identify genes that transiently activate the *mbtB* promoter and has been used in other bacteria to identify genes required during specific phases of *in vivo* mouse and macrophage infection (17, 18). Recombination-based genetic approaches have been also used in mycobacteria to identify genetic regulators of an acid-inducible promoter (19). Using this selection approach in combination with transposon mutagenesis and insertion sequencing (TnSeq), we identified and characterized *mshA*, a mycothiol synthesis gene, and mycothiol as a potential regulator of the *mbtB* promoter in Msm.

## RESULTS AND DISCUSSION

### The Mtb *mbtB* promoter enables Cre-mediated recombination in iron-limited Msm

**FIG 1:**
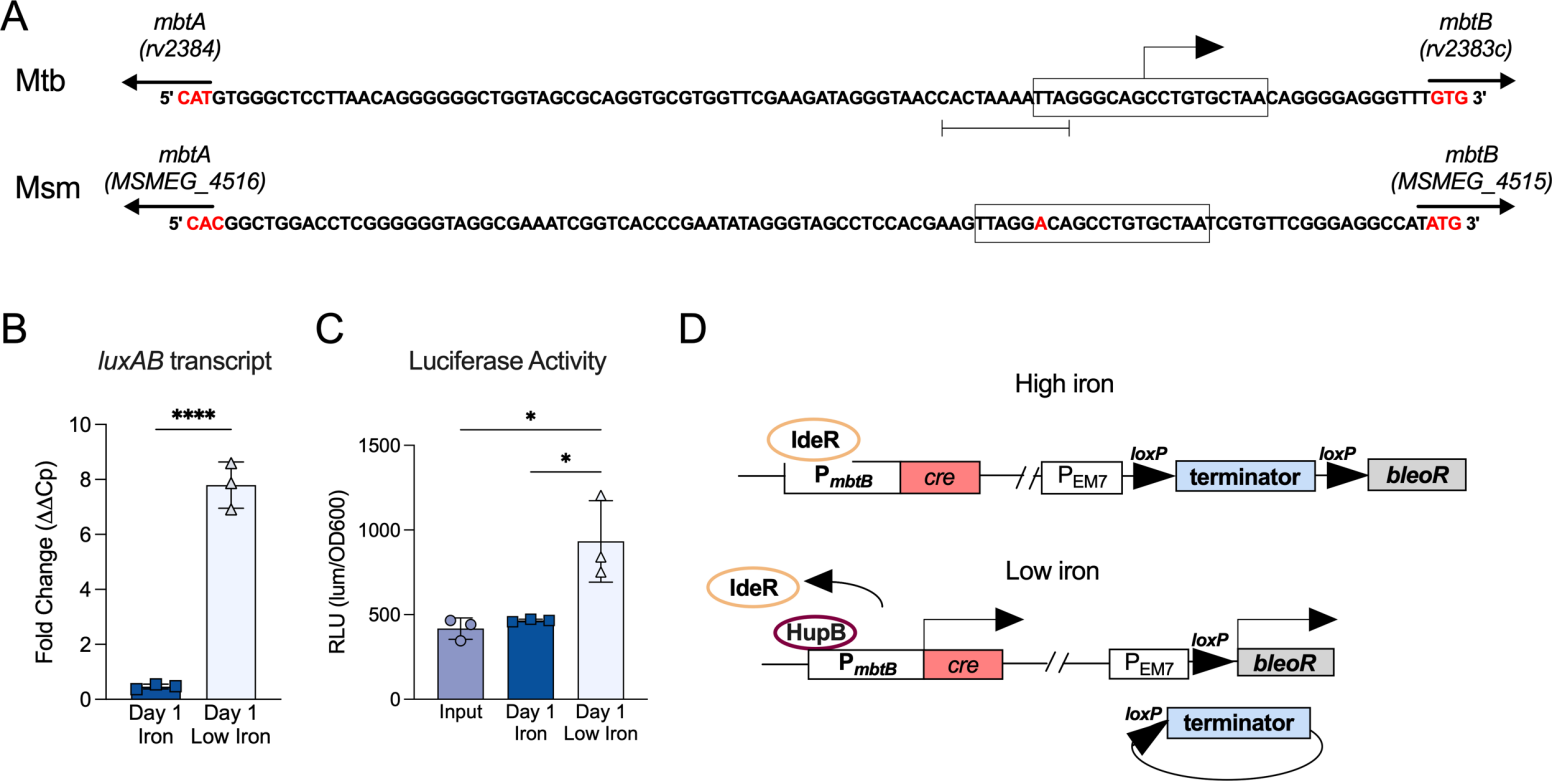
Construction of an iron responsive recombination-based reporter system. (A) Alignment of the *mbtA* and *mbtB* intergenic sequences from Mtb and Msm. Boxes denote IdeR-binding sites, an arrow denotes the transcriptional start site of *mbtB* in Mtb, and brackets highlight the binding site of the positive regulator, HupB (4, 15). The single nucleotide difference between the Msm and Mtb IdeR-binding sites is colored red. (B) Quantification of P*_mbtB_*_-*Mtb*_ driven *luxAB* transcript. Data are normalized to *sigA* mRNA and to mRNA levels at the start of the experiment. Data are means ± SD of three biological replicates ****P < 0.0001 one-way ANOVA with Tukey’s multiple comparison test. (C) Quantification of luciferase activity. Relative luminescent units (RLU) driven by the Mtb P*_mbtB_* promoter upstream of the luciferase *luxAB* operon. Data are represented as mean ± SD of three biological replicates. *P < 0.05 one-way ANOVA with Tukey’s multiple comparison test. Input medium is 7H9 (150 µM iron). (D) Recombination reporter plasmids. The plasmid in the Tweety site (left) contains P*_mbtB-Mtb_* upstream of a *cre* recombinase gene. The plasmid in the Giles site (right) has a terminator sequence flanked by two *loxP* sites situated between a zeocin resistance gene (*bleoR*) and its promoter. When the promoter is active, Cre protein excises the terminator between the *loxP* sites, and the bacteria become zeocin resistant.

We sought to engineer a recombination-based reporter system to identify and select Msm mutants that induce the *mbtB* siderophore gene promoter (P*_mbtB_*). The iron-dependent regulator protein (IdeR) acts as a repressor of *mbtB* transcription in the presence of iron (4), and the IdeR-binding sites are well conserved between Msm and Mtb (Fig. 1A). HupB binding occurs upstream of the IdeR-binding site and positively regulates *mbtB* promoter activity in low iron in Mtb (Fig. 1A) (15). Given the genetic similarity across species, we used the Mtb promoter of *mbtB*, P*_mbtB_Mtb_* to gain insight into its regulation in high iron conditions.

First, we cloned P*_mbtB_* upstream of a luciferase reporter and measured luciferase activity and transcript abundance in low iron conditions. Following 1 day of growth in iron-limited minimal media, *luxAB* RNA transcript levels increased sevenfold and luciferase activity increased twofold, indicating that P*_mbtB_Mtb_* responded to iron starvation in Msm (Fig. 1B and C). We then designed a reporter system that uses P*_mbtB_Mtb_* to drive transcription of the *cre* recombinase gene on a plasmid integrated into the Tweety phage attachment site in the Msm chromosome and introduced a second plasmid into the Giles phage attachment site that contained a terminator sequence flanked by two *loxP* sites situated between a zeocin resistance gene (*bleoR*) and its promoter (Fig. 1D).

**FIG 2:**
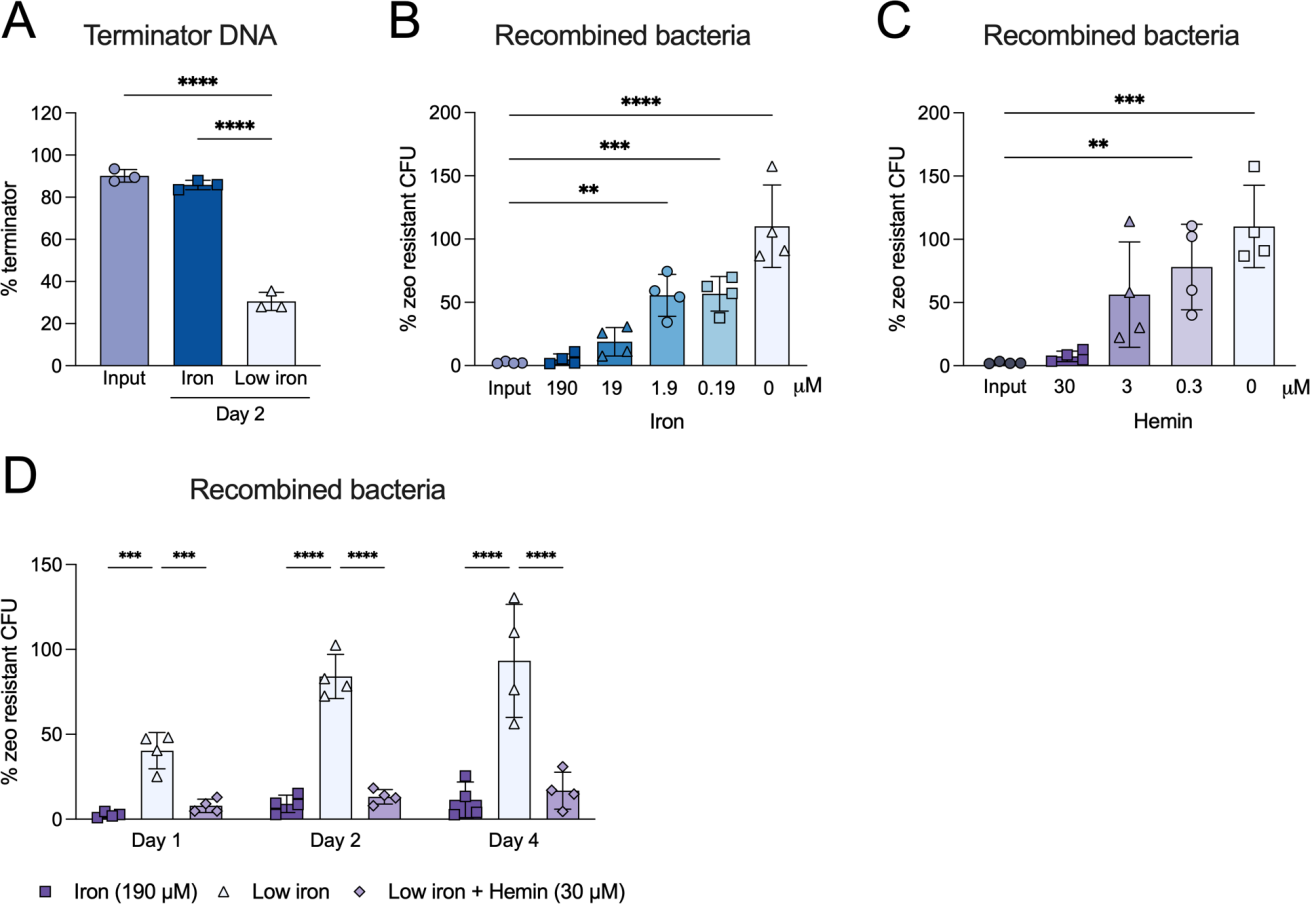
Activity of the iron responsive recombination-based reporter in Msm. (A) Quantification by qPCR of terminator DNA extracted from recombination reporter strains following 2 days of growth in iron (dark blue) or low iron (light blue) media. Input medium is 7H9 (150 µM iron). Iron = Sauton’s medium with 190 µM ferric ammonium citrate. Low iron = chelated Sauton’s medium. Data are normalized to both the integrated plasmid DNA which contains the terminator, as well as a housekeeping gene *sigA*. Data are means ± SD of three biological replicates. ****P < 0.0001 one-way ANOVA with Tukey’s multiple comparison testing. (B and C) Percentage of zeocin resistant bacteria following 2 days of growth in ferric ammonium citrate (B) and hemin (C) at the indicated concentrations. Data are means ± SD of four biological replicates from two independent experiments. **P < 0.01, ***P < 0.005, ****P < 0.0001 two-way ANOVA with Dunnett’s multiple comparison testing. (D) Percentage of recombined bacteria in iron (190 µM), low iron (chelated medium), and low iron media with 30 µM hemin. Data are means ± SD of four biological replicates from two independent experiments. ***P < 0.005, ****P < 0.0001 two-way ANOVA with Tukey’s multiple comparison testing.

In the presence of iron, IdeR is expected to repress P*_mbtB_* resulting in zeocin sensitive bacteria. When iron is limited, IdeR is expected to dissociate from P*_mbtB_*, leading to Cre-*loxP* mediated excision of the terminator sequence (Fig. 1D). This may be facilitated by HupB binding P*_mbtB_* in low iron conditions. Following recombination, the bacteria will become zeocin resistant and can be selected for on zeocin-containing agar. We quantified terminator excision in iron-limited minimal media by quantitative polymerase chain reaction (qPCR). Two days of iron starvation led to a 75% reduction in terminator DNA (Fig. 2A). As expected, the fraction of zeocin resistant Msm increased as the concentration of ferric ammonium citrate or hemin in the culture media decreased (Fig. 2B and C). Further, the fraction of zeocin resistant Msm increased over time during growth in iron-limited minimal media with almost 100% of bacteria becoming zeocin resistant after 4 days (Fig. 2D). The fraction of zeocin resistant Msm was reduced by the addition of 30 µM hemin to iron-limited minimal media, validating that transcription of siderophore biosynthesis genes is repressed when hemin is the sole iron source (20). These data indicate that the Mtb *mbtB* promoter is active in Msm and can drive Cre-mediated recombination and zeocin selection in an iron-dependent manner.

### Transposon mutagenesis identifies mutations that lead to P*_mbtB_* activation in the presence of iron

**FIG 3:**
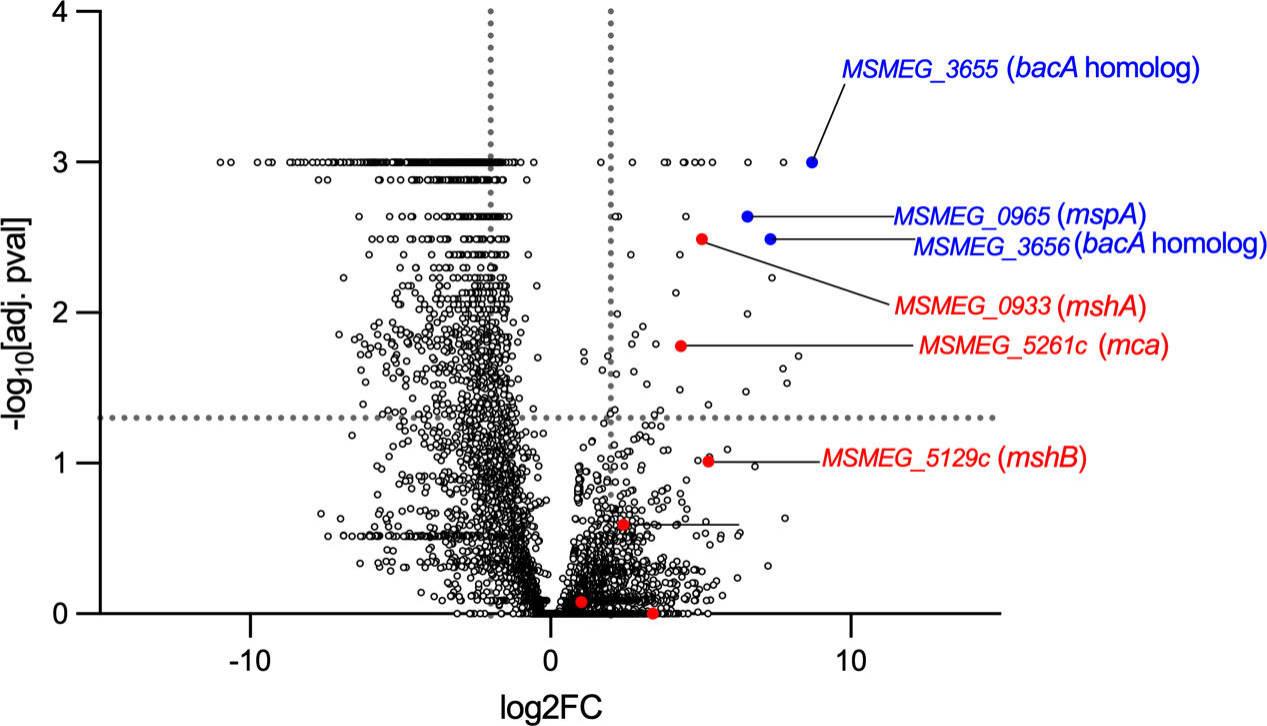
Transposon mutagenesis identifies mutations that lead to P*_mbtB_Mtb_* activation in the presence of iron. Volcano plot depicting depleted and enriched mutants following overnight growth in iron and hemin containing liquid media followed by outgrowth on zeocin containing agar. Mutants with disruption of mycothiol biosynthesis and recycling genes (red), the *mspA* gene (blue) and the homologs of *bacA* (blue) from Mtb are highlighted. The experiment was performed in 7H9 medium supplemented with hemin (30 µM).

The role of the Mtb *mbtB* gene and the activity of its promoter have been well characterized in iron-limited conditions (4, 9, 10). However, sodium dodecyl sulfate and nitric oxide stress have also been shown to lead to *mbtB* transcription in Mtb (11). This was hypothesized to be due to IdeR protein damage and de-repression of P*_mbtB_*. Further, it has been shown that IdeR is required to combat oxidative stress in mycobacteria, as Msm *ideR* mutants are hypersusceptible to H_2_O_2_ (9). When oxidized iron is abundant, the positive regulator HupB may also play a role in regulating oxidative damage by acting as a ferritin-like protein to protect mycobacteria from DNA damage (16). Therefore, we hypothesized that other proteins defending against oxidative stress may regulate *mbtB* promoter activity in high iron conditions, which can generate toxic hydroxyl radicals through Fenton chemistry (12). To test this, we generated transposon libraries in the background of the recombination reporter strain that targeted over 70% of the Msm transposon insertion sites consisting of T and A nucleotides. These libraries were cultured overnight in high iron media containing both iron and hemin. Mutants were then cultured on 7H10 agar containing iron and hemin with kanamycin in the presence and absence of zeocin. Mutants that were depleted and enriched on zeocin containing media were identified using the TRANSIT software (21) (Fig. 3).

As expected, selection on zeocin identified many underrepresented mutants. Several of the highly underrepresented mutants were disrupted in genes with Mtb orthologs whose depletion has been shown to result in increased susceptibility to multiple antibiotics (22). This included mutants with transposon insertions in *MSMEG_4265*c (*mmpS3), MSMEG_0786c (pknG), MSMEG_0788*c (*rv0412*), MSMEG_4240 (*idsA2*), and MSMEG_6183c (*rv3671c*) which may be hypersusceptible to zeocin. Mutants that were enriched by zeocin included those with transposon insertions in *MSMEG_3655* and *MSMEG_3656*, two putative ABC transporters. These genes are orthologs of the Mtb gene *rv1819c* or *bacA*. Mtb lacking BacA is zeocin resistant, though iron uptake is *bacA* independent, and identifying the Msm *bacA* orthologs in our screen validated zeocin selection (23). Mutants with transposon insertions in *MSMEG_0965*, encoding the MspA porin, were also overrepresented on zeocin. Loss of MspA has been shown to result in transcriptional de-repression of the IdeR-regulated exochelin gene *fxbA* in the presence of ferric ammonium citrate, and Msm can acquire iron in a porin-dependent manner in high iron conditions (8). This result confirmed that the TnSeq screen in the recombination reporter strain identified genes whose interruption leads to de-repression of an IdeR-regulated promoter in high iron conditions.

Many overrepresented mutants had transposon insertions in genes involved in oxidative stress response pathways (Table S1). Of note, mutants in the mycothiol synthesis and recycling pathway were overrepresented in our screen (Fig. 3; Table 1). Mycothiol, a low molecular weight thiol, is an important antioxidant in mycobacteria. Msm lacking MshA, which catalyzes the first step of the mycothiol synthesis pathway, has undetectable levels of mycothiol and is hypersusceptible to H_2_O_2_-mediated oxidative damage (24, 25). We therefore sought to determine how mycothiol regulates the IdeR-regulated *mbtB* promoter.

**TABLE 1:** Enrichment of Msm transposon mutants with disrupted mycothiol synthesis and recycling genes after selection with zeocin on agar plates containing iron and heme^*a*^

Open reading frame	Gene	Enzyme	Log_2_FC	Adj. *P* value
*MSMEG_5129*c	*mshB*	N-acetyl-1-D-myo-inosityl-2-amino-2-deoxy-alpha- D-glucopyranoside deacetylase	5.25	0.097
*MSMEG_0933*	*mshA*	Glycosyltransferase	5.04	0.00325
*MSMEG_5261*c	*mca*	Mycothiol S-conjugate amidase	4.34	0.016
*MSMEG_4189*c	*mshC*	Cysteine:1D-myo-inosityl 2-amino-2-deoxy--D-glucopyranoside ligase	3.4	1.0
*MSMEG_4340*	*mscR*	NAD/mycothiol-dependent formaldehyde dehydrogenase	2.43	0.25
*MSMEG_2611*c	*mtr*	NADPH-dependent mycothiol reductase	1.03	0.83
*MSMEG_5783*	*mshD*	N-acetyltransferase	N/A	N/A

^*a*^ Genes are ranked by log_2_FC of the respective transposon mutant. *mshD* (*MSMEG_5783*) is annotated to contain a frameshift that is not a result of a sequencing error. It is considered a pseudogene by TRANSIT sequencing software and was not included in transposon sequencing analysis.

### *MshA* disruption leads to increased *mbtB* promoter activity

**FIG 4:**
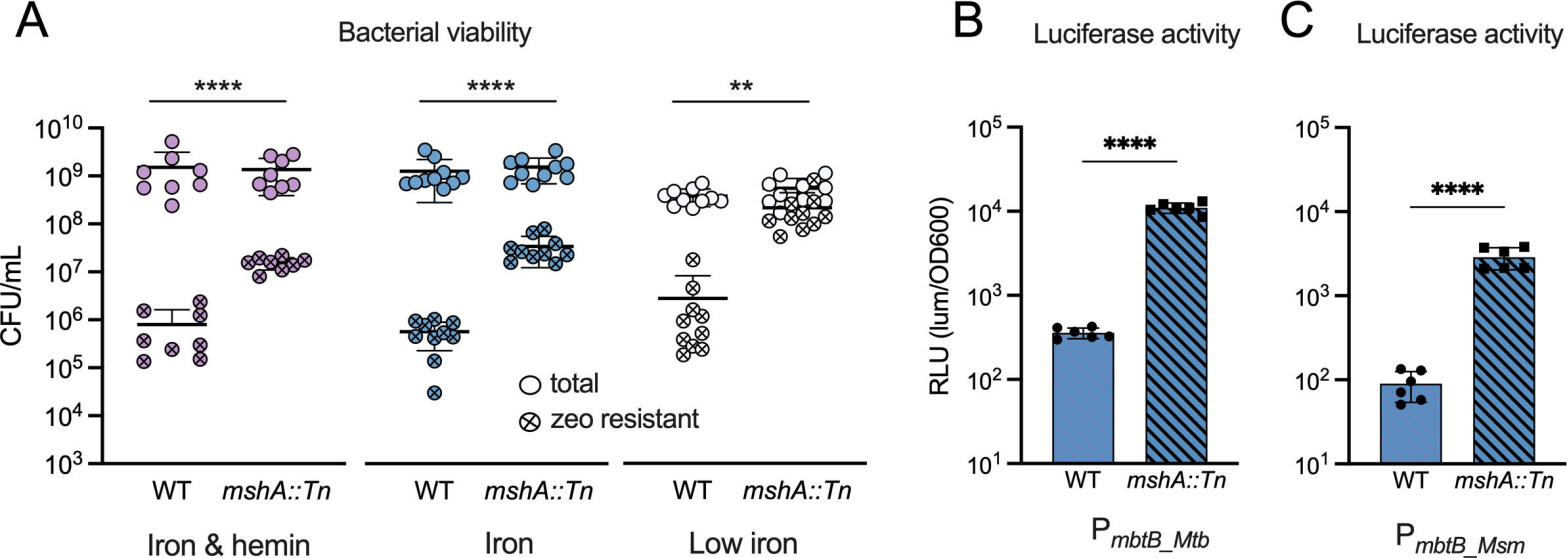
*MshA* disruption leads to increased *mbtB* promoter activity. (A) CFU of WT and *mshA::Tn* mutant enumerated on plates without and with zeocin following 2 days of growth in media with iron (190 µM) and hemin (30 µM), iron alone (190 µM), and low iron (chelated). 8–10 biological replicates for were enumerated over 3–4 independent experiments. Individual data points and means ± SD are shown. Zeocin resistant CFU from WT and *mshA::Tn* were analyzed by unpaired *t*-test ****P < 0.0001, **P < 0.01. (B and C) Luciferase activity in the presence of iron (7H9 medium) of Msm WT and *mshA::Tn* containing the Mtb *mbtB* promoter (P*_mbtB_Mtb_*) (B) and the Msm *mbtB* promoter (P*_mbtB_Msm_*) (C) upstream of the luciferase genes. Data represented as means ± SD of triplicate cultures from two independent experiments. ****P < 0.0001 unpaired *t*-test.

**FIG 5:**
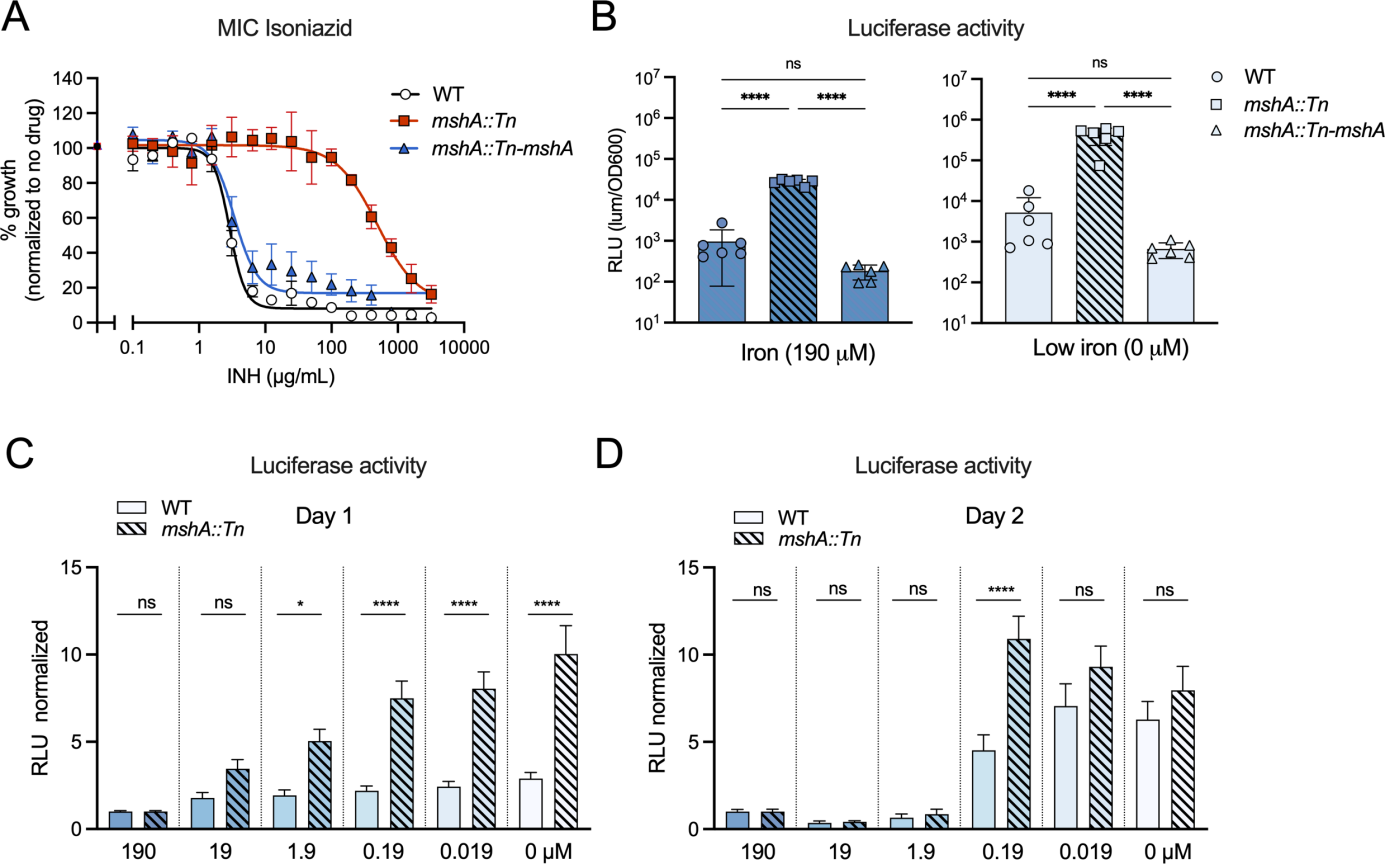
Increased *mbtB* promoter activity in *mshA::Tn* is complemented and time-dependent. (A) Isoniazid (INH) MIC curves of WT, *mshA::Tn* and complemented *mshA::Tn* in 7H9 medium. Data are means ± SEM of five independent biological replicates from two independent experiments quantified after 4 days of bacterial growth. (B) Luciferase activity from the Msm P*_mbtB_* promoter quantified following 1 day of growth in iron and low iron media. Individual data points and means ± SD of six independent biological replicates from two independent experiments are depicted. ****P < 0.0001 using one-way ANOVA with Tukey’s multiple comparison testing. (C) Luciferase activity after 1 day of growth in media with different ferric ammonium citrate concentrations, normalized to RLU in 190 µM iron. Data are means + SEM of nine biological replicates from three independent experiments. *P < 0.05, ****P < 0.0001 using one-way ANOVA with Sidak’s multiple comparison testing. (D) Luciferase activity after 2 days of growth in media with different ferric ammonium citrate concentrations, normalized to RLU in 190 µM iron. Data are means + SEM of nine biological replicates from three independent experiments. ****P < 0.0001 or not significant (ns) using one-way ANOVA with Sidak’s multiple comparison testing.

To validate the TnSeq predictions, we transformed a Msm *mshA::Tn* mutant and the corresponding wild-type (WT) mc^2^155 strain with the recombination reporter (Fig. 1D). Both WT and *mshA::Tn* grew to similar titers in media with different iron concentrations and iron sources (Fig. 4A). We cultured both strains on zeocin-containing agar to identify the fraction of bacteria that underwent recombination and became zeocin resistant. We noticed that the *mc^2^155* parent strain of *mshA::Tn* recombined less efficiently than mc^2^155 from our lab. Notwithstanding, there were 15-fold more zeocin resistant *mshA::Tn* mutant bacteria than zeocin-resistant WT bacteria after 2 days of growth in iron and hemin containing medium consistent with the TnSeq screen (Fig. 4A). Msm *mshA::Tn* also recombined more than WT in the other media, independent of the iron content. Importantly, the minimal inhibitory concentration (MIC) of zeocin is not different for WT and *mshA::Tn* demonstrating that *mshA::Tn* is not intrinsically resistant to zeocin (Fig. S1A). Further, *mshA::Tn* is not zeocin resistant in the absence of the P*_mbtB_-cre* construct (Fig. S1B and C). We further confirmed the increased P*_mbtB_* activity in *mshA::Tn* using the P*_mbtB_*__*Mtb*_ luciferase reporter. Like in the recombination-based reporter system, *mshA::Tn* responded with increased luciferase activity in iron-containing media, relative to the WT (Fig. 4B). Both the recombination and luciferase reporter systems rely on the Mtb *mbtB* promoter and indicate that this promoter is more active in *mshA::Tn* than in WT Msm. We also measured the Msm *mbtB* promoter activity using the luciferase reporter and found that P*_mbtB_Msm_* is also more active in *mshA::Tn* than in WT (Fig. 4C). Complementation of Msm *mshA::Tn* with expression of *mshA* under control of a constitutive promoter on an episomal plasmid reverted the previously reported isoniazid resistance of *mshA::Tn* (Fig. 5A) (26, 27). Similarly, the increased luciferase activity of *mshA::Tn* was fully complemented in iron (190 µM) and iron-limited minimal media culture conditions (Fig. 5B). Thus, inactivation of *mshA* was responsible for the increased P*_mbtB_* activity.

Finally, we determined the kinetics of promoter activation in WT and *mshA::Tn*. We quantified luciferase activity from the P*_mbtB_Msm_-luxAB* fusion in Msm in minimal media following 1 or 2 days of growth in different iron concentrations. To correct for the increased levels of baseline luciferase activity in *mshA::Tn* and to compare changes in luciferase activity over time, relative luminescent unit (RLU) values were normalized to those determined in the presence of 190 µM ferric ammonium citrate for each strain. Luciferase activity in *mshA::Tn* increased significantly more than in WT following 1 day of growth at iron concentrations of 1.9 µM and below (Fig. 5C) and remained high for 2 days in media with 0.19 µM or less iron (Fig. 5D). In contrast, the activity of P*_mbtB_Msm_* in WT Msm increased only after 2 days of iron depletion (in 0.19 mM or less iron) (Fig. 5C and D). Thus, P*_mbtB_Msm_* is more quickly activated at higher iron concentrations in *mshA::Tn* than in WT, suggesting that iron sensing in *mshA::Tn* may be dysregulated.

### Vitamin C reduces promoter activation in *mshA::Tn*

**FIG 6:**
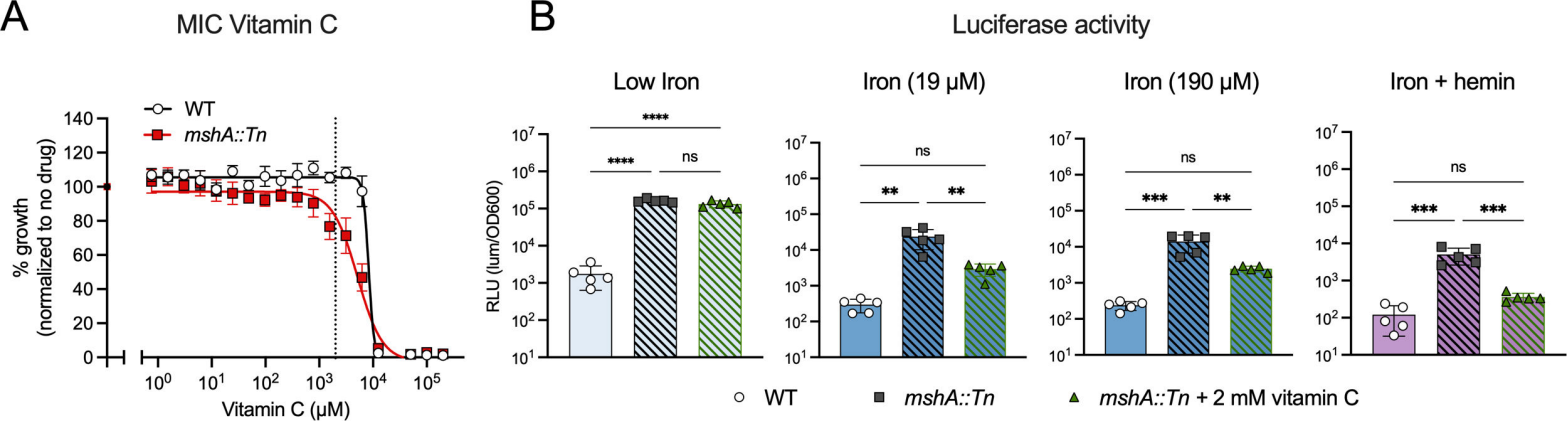
Vitamin C reduces promoter activation in *mshA::Tn* in an iron-dependent manner. (A) Vitamin C MIC curves of WT and *mshA::*Tn in 7H9 medium. Data are means ± SEM of four biological replicates from two independent experiments after 5 days of bacterial growth. Dotted line indicates 2 mM vitamin C. (B) Luciferase activity from the Msm P*_mbtB_* promoter in WT and *mshA::Tn*. Bacteria were grown for 1 day in the indicated media. Iron + hemin medium contains 150 µM iron and 30 µM hemin. *MshA::Tn* was grown with and without vitamin C. Individual data points and means ± SD of five independent biological replicates from two independent experiments are shown. **P < 0.01, ***P < 0.001, ****P < 0.0001, ns = not significant, using one-way ANOVA with Tukey’s multiple comparison testing.

Mycothiol acts as redox buffer and antioxidant, maintaining the reduced state of the mycobacterial cytoplasm (24, 25, 28). IdeR binds two molecules of ferrous (Fe^2+^) iron which facilitates dimerization and binding to DNA (29, 30). The absence of mycothiol may impact the intracellular iron oxidation state leading to the oxidation of ferrous to ferric ion (Fe^3+^) which reduces the DNA-binding capacity of IdeR. To test this hypothesis, we asked if the increased P*_mbtB_* promoter activity in *mshA::Tn* can be reversed by adding vitamin C, which has been shown to promote reduction of ferric to ferrous ion (14, 31). Because vitamin C is toxic to Mtb lacking *mshA* (14) we first determined its MIC against Msm *mshA::Tn* and WT (Fig. 6A). For subsequent experiments, we selected 2 mM vitamin C, which only marginally impaired growth of *mshA::Tn*. Addition of vitamin C to *mshA::Tn* cultured in iron-limited minimal media did not affect P*_mbtB_Msm_* activity as reported by luciferase activity (Fig. 6B). In contrast, after growth in the presence of iron or iron and hemin, addition of vitamin C significantly reduced P*_mbtB_Msm_* activity in *mshA::Tn*. In Mtb, vitamin C has been shown to increase both intracellular and extracellular free iron concentrations (14). It acts as a pro-oxidant and mycothiol-deficient Mtb mutants are exquisitely sensitive to vitamin C, suggesting its effects on the cellular environment are pleiotropic (14). We hypothesize that the reduced P*_mbtB_Msm_* activity in *mshA::Tn* in iron-rich conditions may be the consequence of an altered intracellular redox state affecting the DNA-binding capacity of IdeR. This is consistent with previous work demonstrating that vitamin C downregulated transcription of *ideR* and the siderophore synthesis gene *mbtD* in Mtb (14).

### *MbtB* transcript levels are not consistent with reporter activity

**FIG 7:**
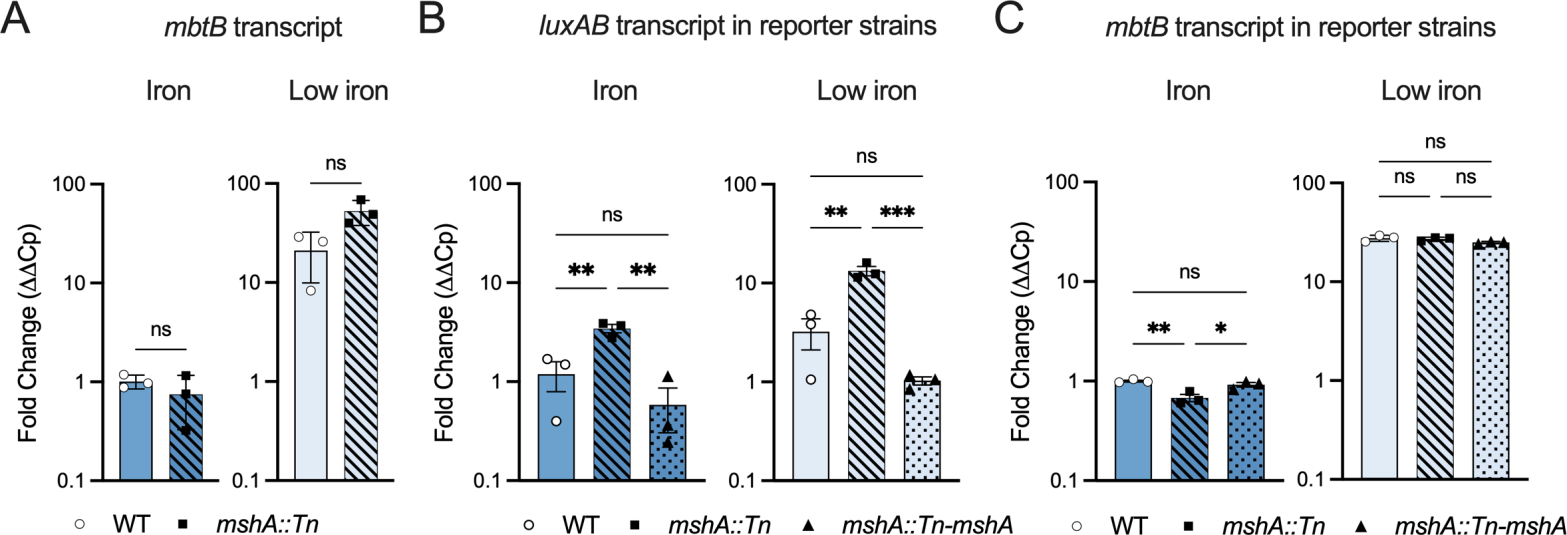
*MbtB* transcript levels are not consistent with P*_mbtB_Msm_* reporter activity. (A) *MbtB* transcript abundance in WT and *mshA::Tn* after growth in iron rich (150 µM) and low iron (chelated) media. Data are means ± SD of three independent biological replicates, ns = not significant using two-tailed *t*-test. (B and C) *LuxAB* transcript (B) and *mbtB* transcript (C) abundance in WT and *mshA::Tn* containing *the* P*_mbtB_Msm_-luxAB* reporter plasmid after growth in iron rich and low iron media. mRNA levels were normalized to *sigA* and to mRNA levels of WT in iron rich media. Data are means ± SD of three independent biological replicates. **P < 0.01, ***P < 0.001using one-way ANOVA with Tukey’s multiple comparison testing.

To determine whether endogenous *mbtB* promoter activity was increased in *mshA::Tn*, we measured *mbtB* transcript levels. Surprisingly, there was no difference in *mbtB* mRNA abundance between WT and *mshA::Tn* in iron replete media (Fig. 7A). Both strains responded to iron restriction with increased *mbtB* transcript levels. We hypothesized that *mshA::Tn* may have acquired mutations that compensate for an altered intracellular redox state. To test this, we transformed the P*_mbtB_Msm_-luxAB* reporter into *mshA::Tn* and measured luciferase mRNA abundance in iron rich and iron depleted media, as we previously demonstrated that luciferase protein activity is increased in the *mshA::Tn* mutant (Fig. 5) . The P*_mbtB_Msm_* driven luciferase transcript level was 3.5-fold higher in *mshA::Tn* than in WT in iron rich media and increased 4-fold in both strains in low iron media (Fig. 7B). However, the endogenous *mbtB* transcript levels of these strains were not higher in *mshA::Tn* compared to WT in iron replete and iron depleted media, although they were almost 30-fold increased in all strains in low iron media (Fig. 7C). This indicates that the endogenous P*_mbtB_Msm_* is regulated differently from P*_mbtB_Msm_* on the integrated reporter plasmid. Although both promoters respond to iron deprivation, P*_mbtB_Msm_* in the attL5 site is more active than native P*_mbtB_Msm_* in iron rich and low iron conditions in *mshA::Tn* relative to WT Msm. The lack of correlation between reporter activity (both luciferase and recombination) and endogenous *mbtB* transcript levels is puzzling. Recently, a discrepancy has been reported between promoter reporter activity and endogenous transcript levels of genes regulated by the nucleoid-associated protein Lsr2 and the authors speculate that the sequence context or changes in the DNA structure may affect access of Lsr2 to DNA (32). Given this, we hypothesize that differences in DNA structure between the endogenous *mbtB* promoter and reporter promoter system may alter binding of *mtbB r*egulatory proteins in our system. While other *mbtB* regulatory proteins, including HupB, have been shown to modify *mbtB* transcription (15), this site was included in our promoter construct directly upstream of the IdeR-binding box. It is therefore possible that a regulatory region outside of the *mbtB* locus that is not included in our reporter plasmid system may affect *mbtB* transcription or mRNA stability, allowing for discrepancies in mRNA transcript levels between the promoter and endogenous gene transcription.

## MATERIALS AND METHODS

### Bacteria and culture media

Msm mc^2^155 was from the American Type Culture Collection. Yossef Av-Gay at the University of British Columbia provided *mshA::TnA1* and the corresponding mc^2^155 WT. Bacteria were cultured at 37°C in Middlebrook 7H9 medium (BD Difco) containing 0.2% glycerol, 0.05% tyloxapol, 0.2% dextrose, 0.5% bovine serum albumin (Roche), and 0.085% NaCl. For quantification, cultures were serially diluted and cultured on Middlebrook 7H10 agar (BD Difco) containing 0.5% glycerol and 10% oleic acid, albumin, dextrose and catalase (OADC) supplement (BD). Zeocin was used at 25 µg/mL, hygromycin at 50 µg/mL, and kanamycin at 25 µg/mL. 7H9 medium was supplemented with 30 µM hemin where indicated, including in transposon mutagenesis experiments. For growth in different iron concentrations, Sauton’s minimal medium was chelated for 2 days with 10 g/liter of BioRad Chelex 100 resin, and when indicated, supplemented with ferric ammonium citrate (190 µM) or hemin (30 µM). Iron-limited minimal medium refers to chelated Sauton’s minimal medium, in which no iron source is added, alternatively referred to as 0 µM iron. For reference, 7H9 medium contains 150 µM ferric ammonium citrate.

### Construction of plasmids

Recombination, luciferase, and complementation plasmids were cloned using the Gateway Cloning Technology (Life Technologies) protocol. The *mbtB* promoter plasmids (*mbtB-cre* and *mbtB-luxAB*) contain the Tweety chromosomal integration site; the zeocin reporter plasmid contains the Giles chromosomal integration site, and the *mshA* gene complementation plasmid is episomal.

### Transposon mutagenesis

A transposon mutant library was generated in the recombination-based reporter strain utilizing Himar1 mutagenesis as previously described (33). Briefly, mid-log phase culture was incubated with MycoMarT7 phage at a multiplicity of infection of 20 at 37°C overnight. The cultures were then washed and plated on 7H10 agar supplemented with 10% OADC, 0.5% glycerol, 0.05% tyloxapol, hygromycin/streptomycin (to select for reporter plasmids), kanamycin (to select for phage integration), and 30 µM hemin. Plates were incubated for 3 days at 37°C, and then DNA was extracted from the colonies, sequenced, and analyzed to determine library coverage (21). To identify mutants overrepresented on zeocin, we incubated the reporter library for 18 h in 7H9 medium with 30 µM hemin and then cultured 10^5^ bacteria per plate on 7H10 plates with 30 µM hemin and either kanamycin or kanamycin and zeocin. Genomic DNA was extracted and sequenced, and TRANSIT resampling was used for analysis (21).

### Luciferase assays

Mycobacterial strains were inoculated from frozen glycerol stocks and grown in with appropriate antibiotics in standard 7H9 medium. Strains were diluted to a starting optical density (OD) of 0.01 in 5 mL media. At the time of the assay, 100 µL bacteria were added to a clear 96-well plate for OD measurement, and 100 µL bacteria were added to a black 96-well plate to measure luciferase activity. Luciferase activity was measured using 10 µL of 1% decanal in molecular grade ethanol. RLU is calculated as the luminescence divided by OD measurement at 600 nm.

### Quantitative reverse transcriptase PCR (qRT-PCR)

A 5M guanidine thiocyanate solution (GTC Sigma-Aldrich Cat. #5140) was added at a 1:1 volume to bacterial cultures followed by centrifugation for 10 min at 4°C. Bacterial pellets were re-suspended in Trizol and bead beaten to release intracellular contents. RNA extraction was performed using the Zymo Research Clean and Concentrator kits (Zymo research Cat. # R1015). qPCR was performed using the Roche Lightcycler 480 and Roche Mastermix reagents. Cp counts quantified using the second derivative maximum and normalized to Msm *sigA*. Primer and probe sequences are available upon request.

### MIC assays

Msm strains were inoculated from frozen glycerol stocks and grown with appropriate antibiotics in 7H9 medium for 48 h at 37°C. Bacteria were diluted to an OD of 0.01 in 7H9 and 200 µL bacteria were added to a 96-well plate. The HP D300e digital dispenser was used to dilute antibiotics in dimethyl sulfoxide (INH) or in 0.1% TritonX in water (zeocin) and deliver antibiotic to plate wells. Vitamin C MICs were performed by diluting L-ascorbic acid (Sigma Aldrich Cat. #A92902) in 7H9 medium and filter sterilizing the solution. Bacteria were grown in standing cultures at 37°C and OD was measured on day 4 or day 5 following treatment.

## SUPPLEMENTAL MATERIAL

Fig. S1 spectrum.00487-24-s0001.docx The *mshA::Tn* mutant is not intrinsically zeocin resistant.

Table S1 spectrum.00487-24-s0002.xlsx TnSeq data of the of the iron responsive *M. smegmatis* reporter strain.

ASM does not own the copyrights to Supplemental Material that may be linked to, or accessed through, an article. The authors have granted ASM a non-exclusive, world-wide license to publish the Supplemental Material files. Please contact the corresponding author directly for reuse.
