# Supplementary material for: Interruption of mycothiol synthesis and intracellular redox status impact iron-regulated reporter activation in Mycobacterium smegmatis
Source: Microbiol Spectr. 2024 Jun 11;12(7):e00487-24. doi: 10.1128/spectrum.00487-24 (PMC11218476; doi:10.1128/spectrum.00487-24)
Supplement: Fig. S1 — The mshA::Tn mutant is not intrinsically zeocin resistant. [file spectrum.00487-24-s0001.docx]

**Supplemental Figure 1. The *mshA::Tn* mutant is not intrinsically zeocin resistant.**

**A.** Zeocin MIC curves of WT and *mshA::Tn*. Data are means +/- SEM of three independent biological replicates quantified after four days of bacterial growth in 7H9 medium. **B.** WT and *mshA::Tn* containing the plasmid with a terminator between the zeocin-resistance gene and its promoter were grown on agar plates with and without zeocin. **C.** WT and *mshA::Tn* containing both recombination reporter plasmids were grown on agar plates with and without zeocin. All experiments and transformations were performed in media containing iron and bacteria were selected on plates containing iron. Plates were imaged after three days of growth.
